# Characterization of cancer survivors clustered by subjective and objective cognitive function scores

**DOI:** 10.1002/cam4.7255

**Published:** 2024-06-21

**Authors:** Taichi Goto, Leorey N. Saligan, Paul Juneau, Stephen G. Gonsalves, Carielle Joy Rio, Letitia Y. Graves, Diane Von Ah

**Affiliations:** ^1^ National Institute of Nursing Research National Institutes of Health Bethesda Maryland USA; ^2^ National Institutes of Health Library National Institutes of Health Bethesda Maryland USA; ^3^ University of Texas Medical Branch School of Nursing Galveston Texas USA; ^4^ The Ohio State University College of Nursing Columbus Ohio USA

**Keywords:** cancer survivorship, cancer‐related cognitive impairment, clustering, VARCLUS™

## Abstract

**Background:**

Cancer‐related cognitive impairment (CRCI) is a prevalent condition that significantly impacts the quality of life of individuals who receive cancer treatment. Clinical management of CRCI presents challenges due to the absence of a standardized assessment. This study identified clinically relevant phenotypic clusters of CRCI based on subjective and objective cognitive function scores.

**Methods:**

In this cross‐sectional study, participants were clustered using the VARCLUS™ based on subjective cognitive impairment assessed through the PROMIS® version 1.0 short‐form subscales of cognitive abilities and cognitive concerns and the CANTAB Cambridge Cognition® scores, which included measures of visuospatial working memory capacity, visual episodic memory, new learning, working memory, executive function, and sustained attention. Each cluster's characteristics were described using demographics, physical and psychosocial factors (physical function, affect, optimism, and social support), and psychoneurological symptoms (anxiety, depression, fatigue, neuropathic pain, and sleep disturbance).

**Results:**

We obtained five clusters from a total of 414 participants, where 99% were female, and 93% were self‐reported white. Clusters 4 and 5 showed the highest PROMIS® cognitive abilities and the lowest measures of cognitive concern, while Clusters 1 and 2 showed the lowest cognitive abilities and the highest cognitive concerns. Clusters 4 and 5 had higher education, income, employment, and higher scores in physical function, positive affect, optimism, and social support. Additionally, individuals in these clusters were less prone to experience severe cancer‐related psychoneurological symptoms.

**Conclusion:**

Our clustering approach, combining subjective and objective cognitive function information, shows promise in identifying phenotypes that hold clinical relevance for categorizing patient presentation of CRCI and facilitating individualized management strategies.

## 1 INTRODUCTION

Cancer‐related cognitive impairment (CRCI) is a common complaint among cancer survivors.^1^ Cancer and its treatments are associated with various types of CRCI, including deficits in memory and processing speed.^2^, ^3^ The most disruptive period of CRCI is the period following cancer treatment and frequently affects a patient's ability to return to work.^4^, ^5^, ^6^ The management of CRCI is challenging because there are no guidelines for its diagnosis or established standards of care.^7^, ^8^

The assessment of cognitive function in cancer survivors is conducted using both subjective and objective measures. Subjective measures, such as self‐reported cognitive impairment, are often used to refer patients for more comprehensive neuropsychological assessments. These subjective measures help identify subtle signs of cognitive impairment that may not always be detected by objective neuropsychological exams.^4^, ^9^, ^10^ Objective neuropsychological assessments commonly employ performance‐based tests to evaluate different domains of cognitive function. These assessments are widely regarded as the gold standard for assessing cognitive impairment.^11^, ^12^ Nonetheless, there exists a discrepancy in correlating subjective self‐reports and outcomes from objective cognitive function assessments.^13^, ^14^

To address this gap, the current study sought to identify clinically relevant phenotypic clusters of CRCI using both subjective and objective cognitive function scores. In addition, phenotypic characteristics unique to each CRCI cluster were described using their associated demographic profiles, physical and psychosocial factors, and psychoneurological symptoms. Ultimately, a phenotypic approach that utilizes commonly obtained clinical information to categorize the subjective and objective presentations of CRCI would facilitate the identification of at‐risk patients for individualized management.

## 2 METHODS

### 2.1 Study participants and ethical considerations

This analysis was part of a parent cross‐sectional study (ClinicalTrials.gov Identifier: NCT04611620) investigating factors related to CRCI in breast and colorectal cancer survivors. These cancers are among the top four most prevalent cancers in the United States and previous research has indicated that up to 75% of breast cancer and 45% of colorectal cancer survivors express cognitive concerns after cancer treatments are completed.^15^, ^16^ Approval for this study was granted by both a Midwest comprehensive cancer center and the Institutional Review Board (IRB) of Indiana University, under protocol number 2009676157. Recruitment was carried out using convenience sampling with IRB‐approved advertisement strategies, including online flyer distribution through cancer‐affiliated resource organizations like Pink Ribbon Connection, Dr. Susan Love Foundation, and Colorectal Cancer Alliance.^17^

Potential participants were directed to a RedCap link survey with eligibility criteria attached. The eligibility checklist was a self‐report questionnaire to enroll individuals aged 21 years or older, who were at least 6 months post‐adjuvant therapy and neo‐adjuvant therapy for early‐stage (Stage I–III) breast or colorectal cancer (current Aromatase Inhibitors or Tamoxifen use at the time of enrollment was allowed) and were experiencing CRCI. Written, online informed consent was obtained from all participants before any study procedures were conducted.

### 2.2 Study measures

The study participants were asked to complete a secure, individualized battery of self‐report questionnaires using the Research Electronic Data Capture (REDCap®) platform. Demographic information such as sex and race was self‐reported. A full description of the measures can be found in the Supplementary Materials.

#### 2.2.1 Cognitive function

##### 2.2.1.1 Subjective cognitive function assessment

Perceived cognitive impairment was assessed using the Patient‐Reported Outcomes Measurement Information System (PROMIS®) version 1.0 short‐form subscales of cognitive abilities and cognitive concerns, each containing eight items.^18^ Higher scores in cognitive abilities items reflect higher cognitive capacity, while higher scores in cognitive concerns items indicate increased cognitive concerns. The test–retest correlation coefficients have been previously reported as 0.80 for cognitive abilities and 0.83 for cognitive concerns,^19^ and Cronbach's alpha was above 0.77 in our previous intervention study,^20^ indicating a high level of consistency in the measured constructs over time. The directions and ranges of scores are available in Supplementary Table S1.

##### 2.2.1.2 Objective cognitive function assessment

Cognitive performance was assessed through remote administration of the CANTAB® (Cambridge Cognition) neuropsychological assessment, which was conducted online. Based on previously reported deficits in various cognitive domains for breast and colorectal cancer survivors, such as episodic memory and learning, sustained attention, working memory, and executive function,^21^ we used the following domains in the CANTAB®, Cambridge Cognition®: (1) visuospatial working memory capacity measured by the CANTAB® – SSPFSL; (2) visual episodic memory and new learning measured by the CANTAB® – PALTE; (3) working memory and executive function measured by the CANTAB® – SWMBE468; and (4) sustained attention measured by the CANTAB® – RVPA and RVPPFA. The directions and ranges of scores are available in Supplementary Table S1.

#### 2.2.2 Physical function, affect, personality, and social support

Physical function was evaluated using the Physical Functioning‐10, a subscale of the Medical Outcomes Study 36‐item Short Form Health Survey (SF‐36).^22^ Higher scores on this subscale indicate worse physical function. Positive and negative affect were evaluated using the Positive and Negative Affect Schedule (PANAS) to assess experiences of each mood during the past week.^23^ Dispositional optimism was assessed using the 10‐item Life Orientation Test‐Revised, with higher scores indicating greater levels of optimism.^24^ Perceived social support was assessed using the Medical Outcomes Study–Social Support Survey (MOS‐SSS).^25^ Higher scores on this survey indicate greater perception of emotional support. The directions and ranges of scores are available in Supplementary Table S2.

#### 2.2.3 Psychoneurological (PN) symptoms

Anxiety,^26^ depression,^26^ fatigue,^27^ neuropathic pain, and sleep disturbance^28^ were evaluated using the PROMIS® short forms specifically designed to assess each respective construct. Bodily pain was measured using the SF‐36.^22^ Higher scores on this subscale indicate greater symptom severity or worse symptomology in terms of bodily pain. Similarly, higher scores on the respective questionnaires indicate more pronounced symptomatology for anxiety, depression, fatigue, neuropathic pain, and sleep disturbance. The directions and ranges of scores are available in Supplementary Table S3.

### 2.3 Establishing clusters and post‐clustering statistical analysis

**FIGURE 1:**
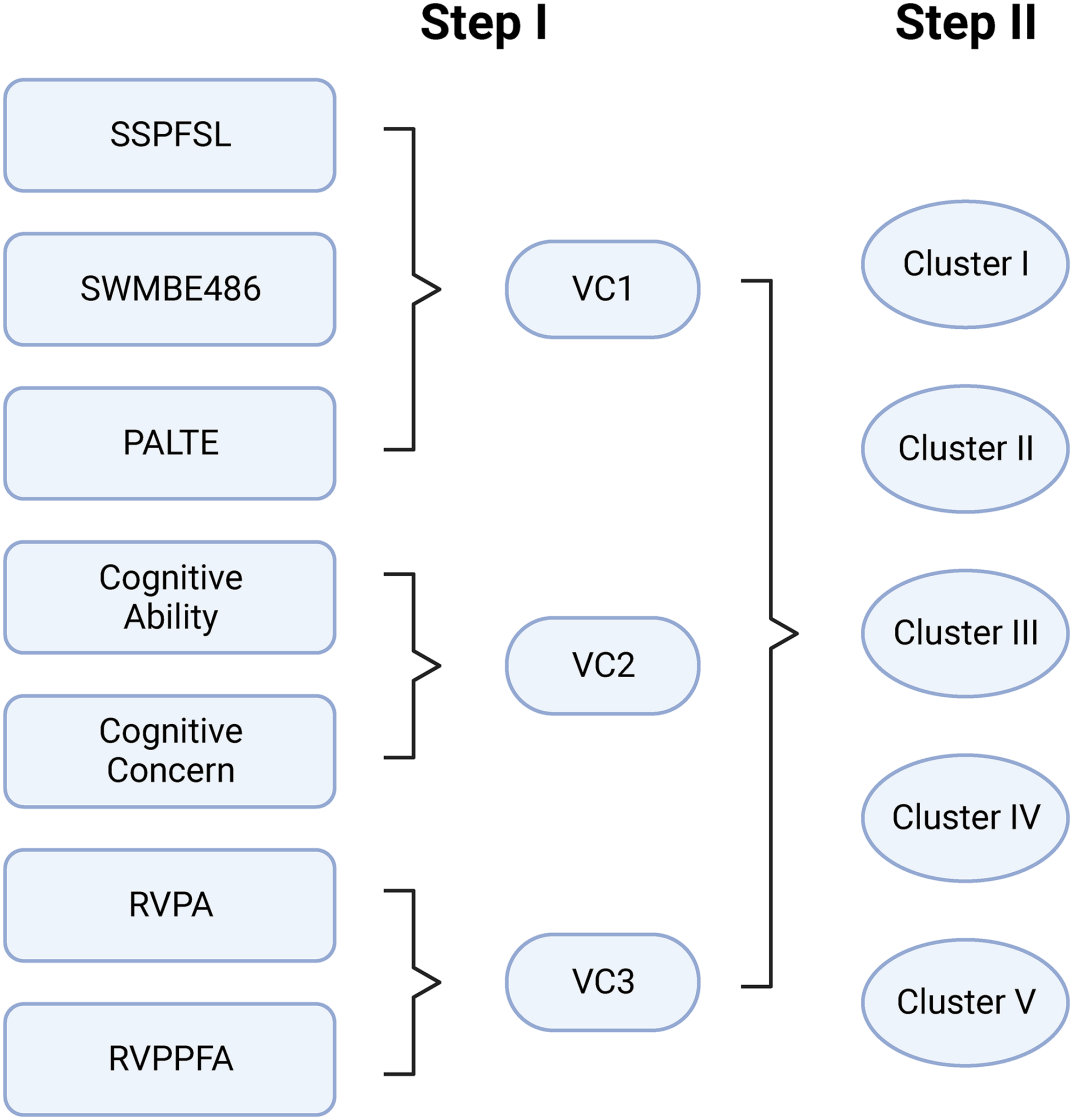
Cluster analyses. The clustering of the study participants was carried out in two steps. In the first step, the seven individual patient scores (1) SSPFSL, CANTAB®—visuospatial working memory capacity; (2) SWMBE468, CANTAB®—Working memory and executive function using; (3) PALTE, CANTAB®visual episodic memory and new learning using; (4) cognitive Abilities, the Patient‐Reported Outcomes Measurement Information System (PROMIS®) short‐form subscales of cognitive abilities; (5) cognitive Concerns, PROMIS® short‐form subscales of cognitive concerns; (6) RVPA, CANTAB®—sustained attention; (7) RVPPFA, CANTAB®—sustained attention were replaced by a principal components‐based procedure by three variable clusters (VC). Values of VC1‐VC3 were then used to create five clusters of study participants in the second step.

The scores of seven cognitive function instruments were analyzed using a variable clustering technique known as the VARCLUS™, developed by the SAS Institute. These instruments encompassed the two perceived cognitive impairment scores (subjective) and five CANTAB® scores (objective) described above. In brief, this is a method of reducing dimensions that replaces individual instrument scores with a summary value obtained through iterative principal components estimation for each participant in the study. Applying the 75% of the variation cutoff, three variable clusters (VC1–3) were used. The variable clusters suggested by the program were then used to perform a *k*‐means clustering of the patients by these surrogate cognitive instrument scores. The analyst examined various scenarios, ranging from three to eight clusters of patients, and selected the one deemed optimal based on the maximum value of the cubic clustering criteria (CCC). A graphic representation of the analysis used in the investigation is described in Figure 1.

After clustering, patient profiles were compared among the clusters using either the use of the analysis of variance (ANOVA) or the Kruskal–Wallis test. Each outcome was then compared pairwise with the others, adjusting for the multiplicity of testing using a false discovery rate. Any *p*‐values less than 0.05 were considered statistically significant. Some categorical variables in the profiles—such as education history, employment status, and income—were shown in heatmaps, which were scaled by row using the “pheatmap” R package. The post‐clustering statistical analyses were performed using R statistical software (version 4.2.1). A sample size calculation and power analysis were not conducted for this sub‐analysis, as this study is intended to generate hypotheses and involves secondary analysis of data already collected from the parent study.

## 3 RESULTS

### 3.1 Study participants and clusters

This analysis was conducted on a total of 414 study participants, with a median age of 55.0 years (interquartile range 13). The majority of the participants were female (99%) and self‐identified as White (93%) (Table 1).

**TABLE 1:** Demographic and clinical characteristics of cluster groups.

		*N* = 414	Clusters				
			1 (*n* = 59)	2 (*n* = 59)	3 (*n* = 102)	4 (*n* = 92)	5 (*n* = 102)
Age (years), mean (SD)		55.3 (9.8)	54.0 (11.0)	53.0 (10.5)	54.5 (14.0)	60.0 (13.5)	55.0 (15.0)
Years since cancer diagnosis (years), median (IQR)		4 (6)	3 (2.75)	5 (5)	4 (6)	4 (5.25)	4 (6)
Sex	Female	408 (99)	59 (100)	58 (98)	102 (100)	90 (98)	99 (97)
Race	White	386 (93)	57 (97)	49 (83)	97 (95)	88 (96)	95 (93)
	Others	28 (7)	2 (3)	10 (17)	5 (5)	4 (4)	7 (7)
Marital Status	Living with someone	308 (74)	36 (61)	43 (72)	82 (78)	69 (75)	78 (77)
	Others	106 (26)	23 (39)	16 (28)	20 (22)	23 (25)	24 (23)
Education History	Highschool/Undergraduate/Associate	268 (65)	45 (76)	44 (75)	72 (71)	56 (61)	51 (50)
	Master's/PhD	146 (35)	14 (23)	15 (25)	30 (29)	36 (39)	51 (50)
Employment Status	Working	271 (65)	39 (66)	36 (61)	75 (74)	48 (52)	73 (72)
	Retired	100 (24)	8 (14)	12 (20)	15 (15)	39 (42)	26 (25)
	Unemployed/Others/Prefer not to answer	43 (10)	12 (20)	11 (19)	12 (12)	5 (5)	3 (3)
Income	> $75 K	210 (51)	23 (39)	33 (56)	58 (57)	40 (43)	56 (55)
	< $75 K	152 (37)	31 (53)	21 (36)	33 (32)	40 (43)	27 (26)
	Unknown/Prefer not to answer	52 (13)	5 (8)	5 (8)	11 (11)	12 (13)	19 (19)
Cancer Type	Breast cancer	373 (90)	52 (88)	52 (88)	89 (87)	87 (95)	93 (91)
	Colon/rectal cancer	41 (10)	7 (12)	7 (12)	13 (13)	5 (5)	9 (9)
Cancer Stage	I	124 (30)	20 (34)	14 (24)	36 (35)	28 (31)	26 (25)
	II	166 (40)	14 (24)	24 (40)	37 (36)	38 (41)	53 (52)
	III/Unsure	124 (30)	25 (42)	21 (36)	29 (29)	26 (28)	23 (23)
Chemotherapy	Yes	372 (90)	53 (90)	54 (92)	89 (87)	87 (95)	89 (87)
Surgery	Yes	412 (99)	59 (100)	58 (98)	102 (100)	92 (100)	101 (99)
Radiation	Yes	287 (69)	40 (68)	40 (68)	69 (68)	66 (72)	72 (71)

*Note*: Except for age and years since cancer diagnosis, all the other variables are presented as *n* (%). Specific categories of the variables were aggregated to group categories with <5 participants. For example, the race category of “Others” includes “American Indian or Alaskan Native,” “Asian,” “Black,” “More than one race,” “Unknown/Prefer not to answer,” and “Other.” For marital status, “Living with someone” includes “living with partner,” and “married,” while “Others” includes “Divorced,” “Single,” “Widowed,” and “Other/Prefer not to answer.” For education, “Highschool/ Undergraduate/ Associate” category includes “High school graduate,” “Undergraduate/ Bachelor's degree or equivalent” and “Associate's degree/ some college;” while the “Master's/PhD” category includes “Master's degree or equivalent” and “PhD or equivalent.” For employment status, “Working” category includes “Full‐time (>35 h/week),” “Homemaker,” and “Part‐time (<20 h/week).”

The VARCLUS™ technique suggested three groupings of variables as surrogates for the original seven cognitive instrument scores: (1) a component that included the visuospatial working memory capacity (CANTAB®‐SSPFSL), working memory and executive function (CANTAB®‐SWMVE486), and visual episodic memory and new learning (CANTAB®‐PALTE) scores (hereafter, designated as VC1); (2) another that contained the PROMIS® Cognitive Abilities and Perceived Cognitive Concerns scores (VC2); and (3) a third that contained the sustained attention (CANTAB® RVPA & RVPPFA) scores (VC3). The CCC values from *k*‐means clustering were −36.587 for 3 clusters, −39.946 for 4 clusters, −31.915 for 5 clusters, −32.605 for 6 clusters, −38.594 for 7 clusters, and −36.265 for 8 clusters. We selected five patient clusters because the scenario had the highest CCC value among the six scenarios (Figure 1).

**FIGURE 2:**
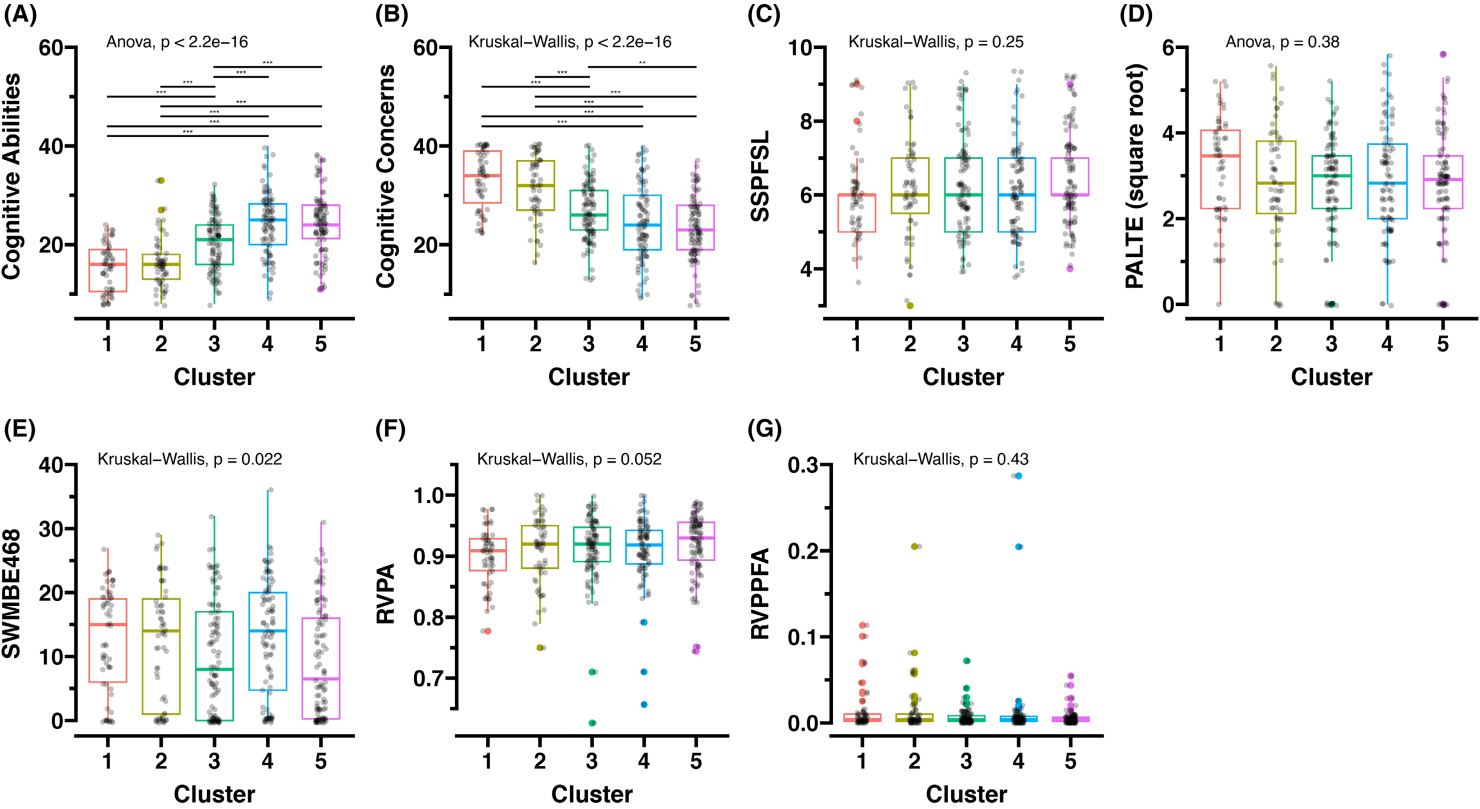
Comparing subjective and objective measures of cognitive function among clusters. The scores were compared by ANOVA followed by pairwise *t*‐tests or the Kruskal–Wallis test followed by pairwise Wilcoxon's rank sum tests, as appropriate. (A) The Patient‐Reported Outcomes Measurement Information System (PROMIS®) short‐form subscales of cognitive abilities. (B) PROMIS® short‐form subscales of cognitive concerns. (C) CANTAB®—SSPFSL for visuospatial working memory capacity. (D) CANTAB®—PALTE for visual episodic memory and new learning. (E) CANTAB® – SWMBE468 for working memory and executive function. (F, G) CANTAB® – RVPA and RVPPFA for sustained attention. “**” indicates a *p*‐value less than 0.01, and “***” indicates a *p*‐value less than 0.001.

Clusters 4 and 5 had the highest cognitive abilities (the PROMIS®), while Clusters 1 and 2 had the lowest (Figure 2A). Cognitive concerns (the PROMIS®) were highest in Clusters 1 and 2 and lowest in Clusters 4 and 5 (Figure 2B). No significant difference was found in the five CANTAB® scores (Figure 2C,D,F,G), except for working memory and executive function (CANTAB® – SWMBE468) where post hoc pairwise comparisons did not yield significant differences (Figure 2E). The cognitive function scores for each cluster are available in Table S1.

### 3.2 Demographic and clinical characteristics of each cluster

**FIGURE 3:**
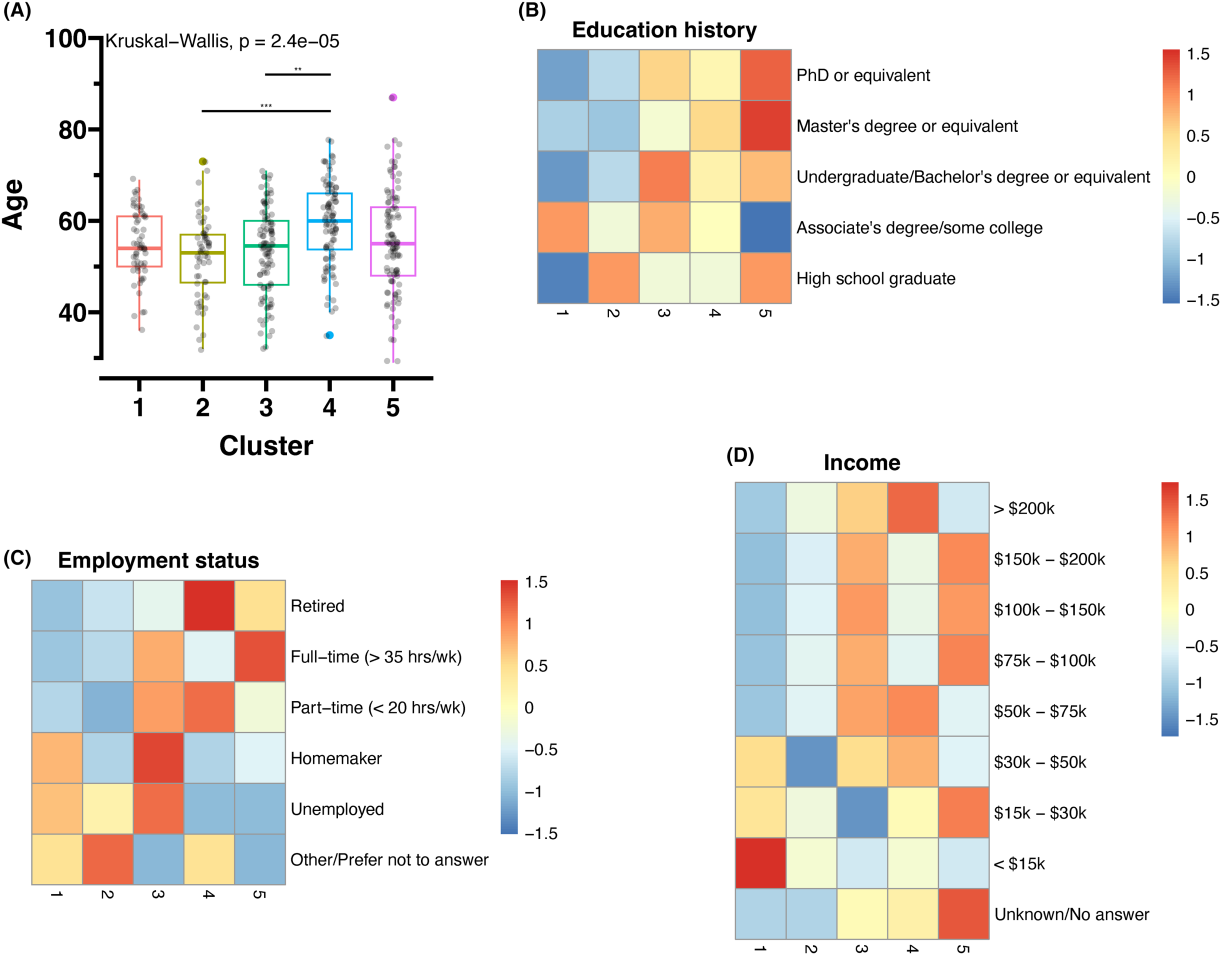
Comparing demographic characteristics among clusters. (A) Median ages were compared among the clusters using the Kruskal–Wallis test followed by Wilcoxon's rank sum test for pairwise comparisons. “**” indicates a *p*‐value less than 0.01, and “***” indicates a *p*‐value less than 0.001. (B–D) A heatmap for education history, employment status, and income, respectively.

Age (Kruskal‐Wallis test, *p* < 0.0001), race (Fisher's exact test, *p* = 0.0030), education history (Fisher's exact test, *p* = 0.0005), employment status (Fisher's exact test, *p* = 0.0005), and income (Fisher's exact test, *p* = 0.0200) significantly differed among the clusters (Table 1). Among the study participants, 90% underwent chemotherapy, 99% underwent surgery, and 69% underwent radiation therapy, which did not differ significantly between clusters. Subsequent post hoc multiple pairwise comparisons revealed a significant age difference, with Cluster 4 being older than Clusters 2 and 3 (Figure 3A). In addition, Clusters 4 and 5 had higher education levels, were more likely to be employed or already retired, and had higher income than Clusters 1 and 2 (Figure 3B–D). More than 70% of those with less than $15 k annual household income were categorized in Cluster 1 (Figure 3D).

### 3.3 Physical and psychosocial characteristics of each cluster

**FIGURE 4:**
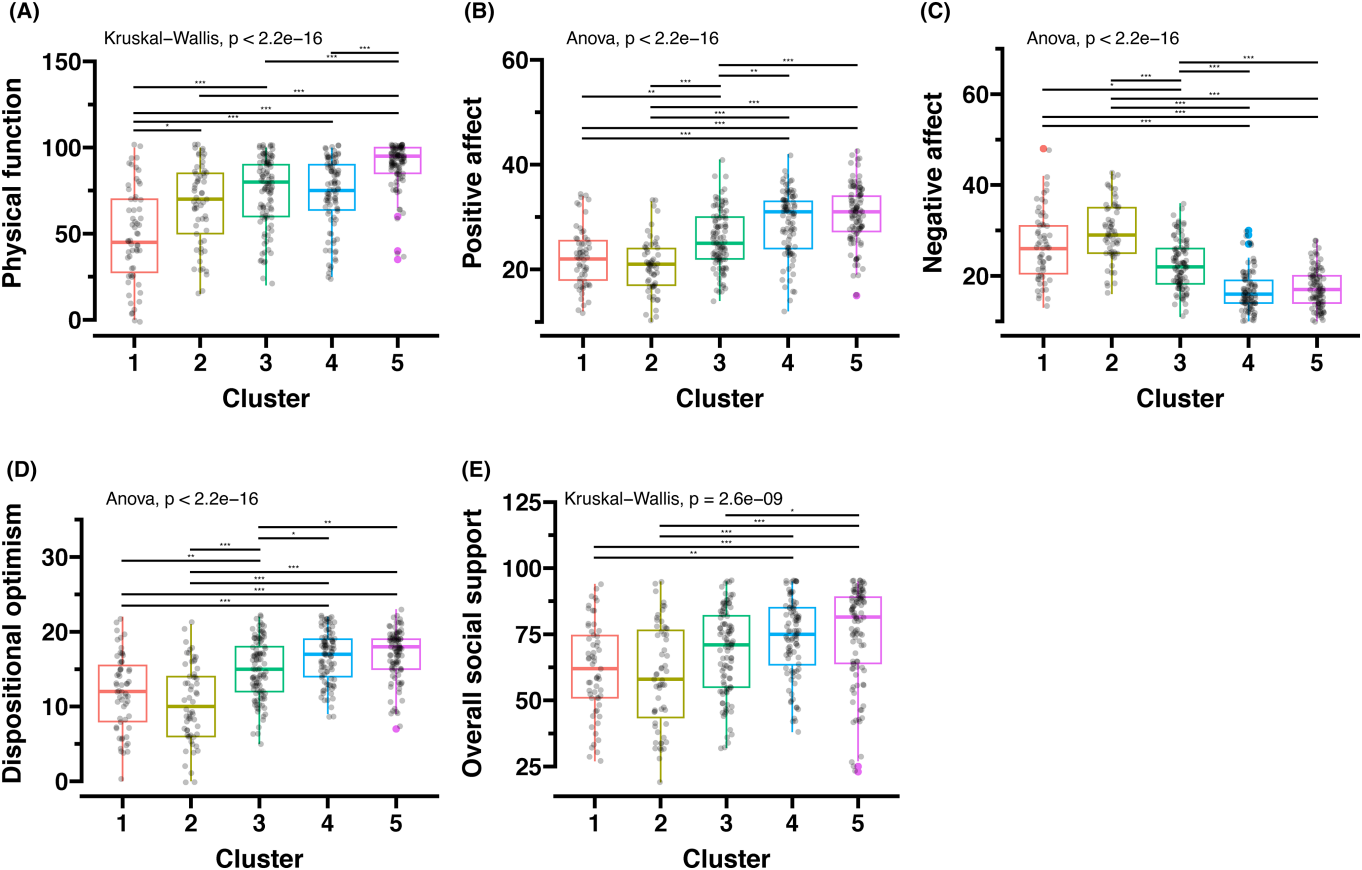
Comparing physical function, affect, personality, and social support scores among clusters. The scores were compared by analysis of variance followed by pairwise *t*‐tests or the Kruskal–Wallis test followed by pairwise Wilcoxon's rank sum tests, as appropriate. (A) Physical function scores using the Physical Functioning‐10, which is a subscale of the Medical Outcomes Study 36‐item Short Form Health Survey. (B, C) Positive and negative affects using the Positive and Negative Affect Schedule (PANAS) Schedule. (D) Dispositional optimism using the 10‐item Life Orientation Test‐Revised. (E) Social support using the Medical Outcomes Study–Social Support Survey (MOS‐SSS). “**” indicates a *p*‐value less than 0.01, and “***” indicates a *p*‐value less than 0.001.

Cluster 5 exhibited the highest level of physical function among the clusters while Cluster 1 had the lowest (Kruskal–Wallis test, *p* < 0.0001) (Figure 4A). Participants in Clusters 4 and 5 exhibited a higher likelihood of having a more positive affect when compared with those in Clusters 1 and 2 (ANOVA, *p* < 0.0001) (Figure 4B). Participants in Clusters 1 and 2 were more likely to have a more negative affect compared to those in Clusters 4 and 5 (ANOVA, *p* < 0.0001) (Figure 4C). Clusters 4 and 5 demonstrated the highest levels of optimism, while Clusters 1 and 2 had the lowest (ANOVA, *p* < 0.0001) (Figure 4D). Regarding social support, participants in Clusters 4 and 5 reported higher levels of social support in comparison with those in Clusters 1 and 2 (Kruskal‐Wallis test, *p* < 0.0001) (Figure 4E). Supplementary Figure S1 provides specific scores for emotional/informational support, tangible support, affectionate support, and positive social interaction within each cluster, while Table S2 lists all the scores of these variables for each cluster.

### 3.4 Symptom phenotypes of each cluster

**FIGURE 5:**
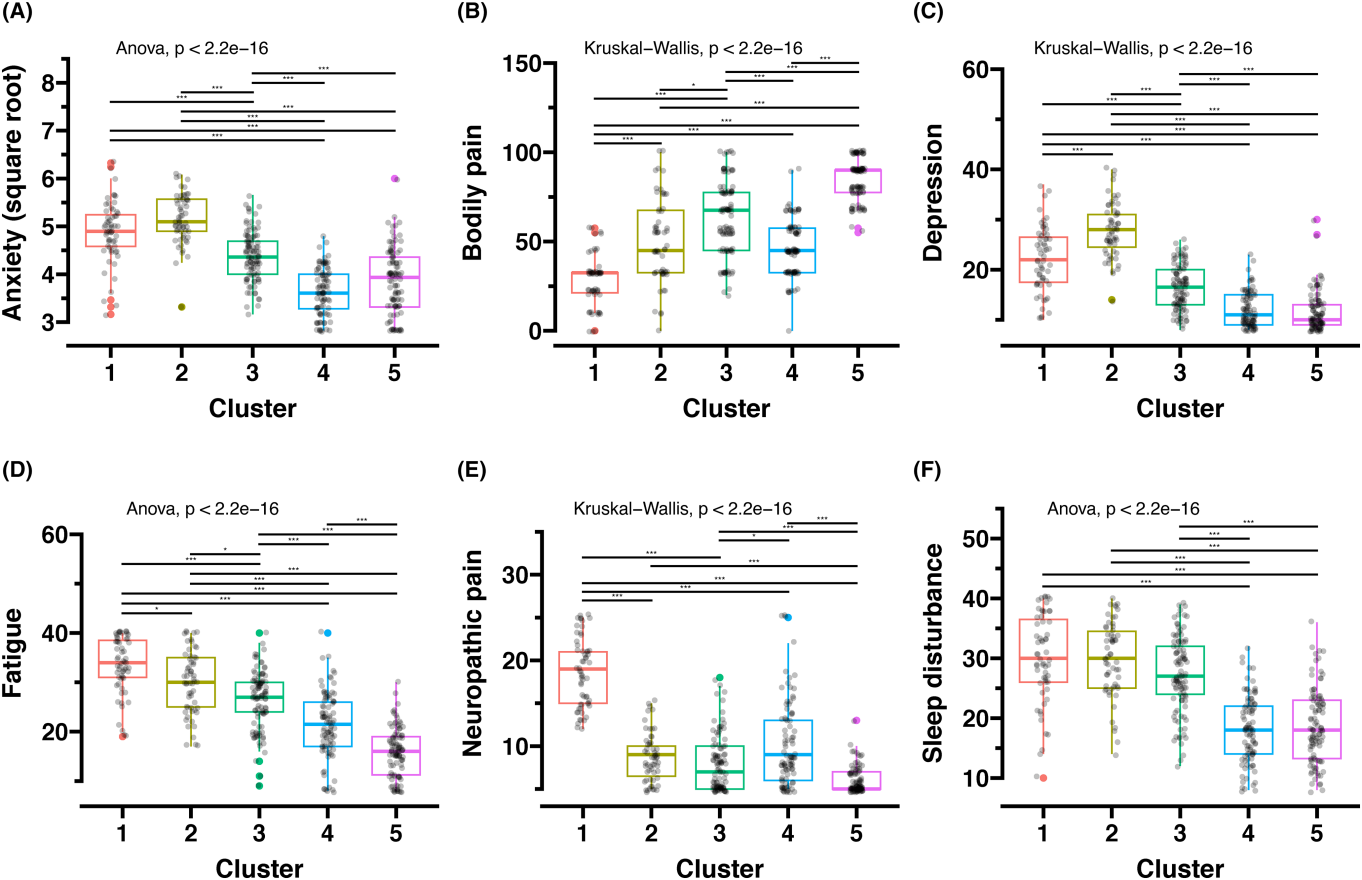
Comparing symptom scores among clusters. The scores were compared by analysis of variance followed by pairwise *t*‐tests or the Kruskal–Wallis test followed by pairwise Wilcoxon's rank sum tests, as appropriate. (A) Anxiety score using the Patient‐Reported Outcomes Measurement Information System (PROMIS®). (B) Bodily pain score using the Medical Outcomes Study 36‐item Short Form Health Survey. (C–F) Depression, fatigue, neuropathic pain, and sleep disturbance using PROMIS®. “**” indicates a *p*‐value less than 0.01, and “***” indicates a *p*‐value less than 0.001.

Figure 5 illustrates each symptom phenotype within the five clusters. Anxiety levels were higher in Clusters 1 and 2 and lower in Clusters 4 and 5 (ANOVA, *p* < 0.0001) (Figure 5A). Bodily pain was most severe in Cluster 5 and mildest in Cluster 1 (Kruskal–Wallis test, *p* < 0.0001) (Figure 5B). Depression was most severe in Cluster 2 followed by Cluster 1, and mildest in Clusters 4 and 5 (Kruskal–Wallis test, *p* < 0.0001) (Figure 5C). Fatigue scores exhibited a gradual decrease from Cluster 1 to Cluster 5, with significant differences observed when comparing each cluster pair within the five clusters (ANOVA, *p* < 0.0001) (Figure 5D). Notably, Cluster 1 had the most severe neuropathic pain among the five clusters (Kruskal–Wallis test, *p* < 0.0001) (Figure 5E). Additionally, Clusters 4 and 5 showed milder sleep disturbance compared to Clusters 1 and 2, which experienced the most severe sleep disturbance (ANOVA, *p* < 0.0001) (Figure 5F). The symptom phenotype scores for each cluster can be found in Table S3.

## 4 DISCUSSION

We identified clinically promising clusters based on both subjective and objective cognitive function assessment scores in breast and colorectal cancer survivors. Important behavioral and social determinants of health emerged as potential targets for optimal management of CRCI. Although a few of these clusters shared similar attributes, such as Clusters 1 and 2 reporting the highest cognitive concerns, while Clusters 4 and 5 having the lowest cognitive concerns, each of these clusters exhibited clear distinctions that could be relevant for practice. For example, individuals in Cluster 4 were more likely to be retired and earning >$200 K, while participants in Cluster 5 had graduate educational degrees, the highest physical function, dispositional optimism, and overall social support, but the worst bodily pain. On the contrary, participants in Cluster 1 were mostly unemployed or homemakers, mostly earning <$15 K, lowest educational attainment, lowest physical function and bodily pain, but highest fatigue and neuropathic pain. Cluster 2 participants had the lowest dispositional optimism and overall social support, but the highest depression and anxiety.

Given the study design, it is not possible for us to establish causal relationships between the identified clusters and their respective phenotypic attributes. However, the distinct phenotypic traits exhibited by each cluster can yield valuable insights, facilitating the development of early, personalized care management strategies for cancer survivors to prevent CRCI. An intervention initiated prior to cancer treatment in patients with high‐risk demographic profiles identified in this study holds promise for mitigating CRCI. These strategies may include exercise and yoga to target physical function, as well as psychological interventions targeting perceived stress.^29^, ^30^, ^31^ However, further validation of these targets and phenotypes through longitudinal studies and clinical trials is warranted.

While our clustering strategy did not identify any significant difference in objective cognitive performance test scores obtained using CANTAB® between the clusters, despite some tendencies for Cluster 1 to have the worst scores, we were able to distinguish between the clusters based on the perceived, subjective cognitive function scores. A systematic review revealed that numerous studies demonstrated a lack of or a weak association between subjective self‐assessment and objective evaluations of CRCI.^32^ Possible explanations for this discrepancy may involve the utilization of less‐sensitive neuropsychological assessments, which may not effectively capture the subtle cognitive alterations often experienced by cancer survivors and, it is also worth noting that self‐reported and objective measures of cognitive function may be assessing different, distinct constructs.^13^ Hence, multiple groups, including this study, use both subjective and objective measurements to capture all domains of CRCI.^33^

The impact of education history, employment status, and income on cognitive impairment has been the subject of extensive investigation. A review of multiple longitudinal cohort studies and meta‐analyses has revealed that educational attainment exerts positive effects on cognitive function.^34^ The author of this review highlighted several factors influencing educational attainment, such as parental resources, social support, and scholastic opportunities. These factors, in turn, may also influence an individual's own socioeconomic resources and environmental exposures throughout adult life, partly by determining occupation, social status, financial resources, and, directly or indirectly, access to quality health care.^34^ Our findings also observed that the participants with higher education levels (e.g., Clusters 4 and 5) were more inclined to be employed and have higher incomes, as well as socially supported than those in other clusters.

Our study also detected an association between cognitive function and physical function. Cognitive impairment was previously associated with higher odds of experiencing low physical function over time in a 20‐year longitudinal cohort study.^35^ Another study revealed a reciprocal relationship between lower physical function, as assessed by gait speed and handgrip strength, and a more pronounced decline in global cognitive function among older individuals.^36^ Specifically, those authors found that loss of executive function was associated with a decline in gait speed.^36^ Our pairwise comparisons did not indicate a significant difference in executive function as measured by SWMBE468. However, our findings suggest that implementing preventive measures addressing both cognitive impairment and physical function could potentially attenuate the future decline in cognitive and physical functions among cancer survivors. This further emphasizes the importance of assessment of both cognitive and physical functions at the very early phase of cancer or its treatment.

The present study also found significant differences in mood and affect between identified clusters. While limited in number, studies examining the impact of positive or negative affect on cognitive impairment have indicated that increased levels of negative affect have been linked to a heightened risk of mild cognitive impairment in women.^37^ Additionally, elevated levels of subjective memory complaints have been reported among healthy older adults in relation to their negative affect.^38^ Optimism was also reported to be linked with subjective cognitive impairment,^39^ and greater optimism was associated with positive affect in cancer survivors.^40^ Our study revealed distinct subjective measurements for cognitive abilities and concerns among clusters. Notably, mood factors, including lower positive affect, higher negative affect, and less optimism, emerged as robust predictors of cognitive impairment in our study participants.

Distressing psychoneurological (PN) symptoms and cognitive impairment are frequently experienced by cancer survivors during and even after treatment, lasting for months or even years.^41^ It is noteworthy that these symptoms often co‐occur and may lead to additional adverse health outcomes.^41^, ^42^, ^43^, ^44^, ^45^, ^46^ Nevertheless, the relationship between PN symptoms and cognitive impairment in cancer survivors remains inadequately explored. In the present study, each PN symptom score was distinctive among the clusters. Notably, the fatigue score was significantly different in each pairwise comparison among the five clusters. Our previous prospective study in prostate cancer survivors showed that increasing fatigue during radiation therapy was associated with worse cognitive deficits and more difficulties performing cognitive tasks than those with stable fatigue.^47^ Similar relationships between cognitive function and fatigue were also observed in various cancer types, such as localized colorectal cancer and breast cancer.^16^, ^48^ While the presence of causal relationships has not been explored, correlations or co‐occurrence of CRCI and depression/anxiety have been reported.^49^, ^50^, ^51^ There is very limited evidence on the biobehavioral factors linking chronic pain with cognitive dysfunction in cancer survivors, but it has been found that HIV patients who had cognitive impairment had a higher prevalence of chronic pain with more neuropathic symptoms in a cross‐sectional study.^52^ Another study using the spared nerve injury neuropathic pain model in mice showed increased CXCL12‐mediated monocytes, which resulted in memory decline. When applied to patients, that study also observed that circulating monocytes and plasma CXCL12 were elevated in chronic pain patients, and both markers were closely correlated with memory decline.^53^ Based on our findings and these previous reports, intensive assessment and management of PN symptoms clustered with cognitive impairment in cancer survivors should be considered as part of the standard of care.

While the associations found in the present study show promise in facilitating diagnosis and care for patients with CRCI, it will be critical to evaluate these factors in a more diverse cohort. We employed social media and utilized online cancer‐affiliated resource sites for recruitment, potentially introducing selection bias. It is crucial to exercise caution when generalizing our findings, as those who accessed our advertisements were more likely to participate. Notably, approximately 99% of our study participants were female, 93% were White, and 90% were diagnosed with breast cancer, imposing a limitation on the interpretation and generalizability of the results. As such, the application of the results in a different sociodemographic setting warrants careful consideration. Given the multiple social and environmental factors associated with CRCI, a more racially diverse sample would be ideal for future investigations. In addition, the PCA methodology used in the VARCLUS™ technique has some potential limitations.^54^ However, as a descriptive or summary measurement, the PCA does not require fidelity to classical assumptions like multivariate normality, as would be the case if we were to use the results of the VARCLUS technique (the PCAs) inferentially.^55^ We did not use the results from the PCA methodology in an inferential way but as a means of summarizing the relationships between the clustering variables or features. These summaries were used in subsequent *k*‐means cluster analysis, where the PCAs' properties potentially had a greater impact on the results.^55^ However, the process of calculating the PCAs from the VARCLUS™ before clustering produced variates with properties that made them more suitable for *k*‐means clustering (e.g., proper scaling) than using the original data.

In conclusion, we have successfully characterized five distinct cognitive function clusters in our cancer survivor cohort by utilizing demographic, medical, psychosocial, physical, and patient‐reported symptom information. Our statistical approach showed a promising phenotypic clustering technique with a concurrent validity that revealed strong associations between the clusters and education history, employment status, income, physical function, and PN symptoms, which has the potential to clinically categorize patients for optimal individualized CRCI management. While this study adopted a cross‐sectional design, it highlighted the influence of behavioral and sociodemographic variables, such as symptoms and educational history, on cognitive performance,^34^, ^56^ emerging as key behavioral and social determinants of health useful in strategizing the appropriate management for cognitive deterioration in cancer survivors. It is still imperative to conduct longitudinal investigations to explore the causal relationships between risk factors and cognitive performance. Such an approach promises to enhance our comprehension of the multifaceted clinical subtypes of CRCI.

## AUTHOR CONTRIBUTIONS

**Taichi Goto:** Formal analysis (equal); investigation (equal); methodology (equal); software (equal); writing – original draft (lead); writing – review and editing (equal). **Leorey N. Saligan:** Conceptualization (equal); funding acquisition (equal); methodology (equal); supervision (lead); writing – review and editing (lead). **Paul Juneau:** Formal analysis (lead); methodology (equal); software (equal); writing – review and editing (equal). **Stephen G. Gonsalves:** Writing – review and editing (equal). **Carielle Joy Rio:** Writing – review and editing (equal). **Letitia Y. Graves:** Writing – review and editing (equal). **Diane Von Ah:** Conceptualization (lead); data curation (lead); funding acquisition (lead); methodology (equal); supervision (equal); writing – review and editing (equal). We thank the NIH Fellows Editorial Board who assisted with editing this article Michael Steele, the data manager of the National Institute of Nursing Research, who assisted in preparing the data for analyses, and Marie Iwaniuk, PhD, a biostatistician of the National Institutes of Health Library, who assisted in statistical analyses.

## FUNDING INFORMATION

This study received support from the Intramural Research Program of the National Institute of Nursing Research, the National Institutes of Health, as well as the Schools of Nursing at Indiana University, and The Ohio State University. The authors bear full responsibility for the content presented in this study, and it is important to note that the views expressed do not inherently reflect the official perspectives of the National Institutes of Health or the National Institute of Nursing Research.

## CONFLICT OF INTEREST STATEMENT

The authors do not have any pertinent financial or non‐financial interests to disclose, aside from those that have been elucidated in the funding section.

## INSTITUTIONAL REVIEW BOARD STATEMENT

Approval for this study was granted by both a Midwest comprehensive cancer center and the Institutional Review Board (IRB) of Indiana University, under Protocol number 2009676157.

## INFORMED CONSENT STATEMENT

Prior to initiating any study procedures, written informed consents were secured from participants online.

## Supporting information


Data S1:

Data S2:

## Data Availability

The datasets analyzed in the present study can be accessible upon reasonable request to the corresponding author.
