# Correction to “Characterization of Cancer Survivors Clustered by Subjective and Objective Cognitive Function Scores”

**DOI:** 10.1002/cam4.71828

**Published:** 2026-04-13

Goto T, Saligan LN, Juneau P, Gonsalves SG, Rio CJ, Graves LY, Von Ah D. Characterization of Cancer Survivors Clustered by Subjective and Objective Cognitive Function Scores. *Cancer Medicine*. 2024;(12):e7255. doi: https://doi.org/10.1002/cam4.7255.

Following publication of the article [1], we became aware that the SF‐36 Bodily Pain measure had been described and interpreted with an incorrect score direction. Specifically, the 2‐item Bodily Pain subscale of the 36‐Item Short‐Form Health Survey (SF‐36) is scored on a 0–100 transformed scale in which higher values represent less bodily pain (better health status) and lower values represent more bodily pain (worse health status) [2]. In the published version, we mistakenly stated/implied that higher Bodily Pain scores reflected greater pain severity, which is opposite to the SF‐36 scoring convention.

This issue is limited to the narrative description and interpretation of the Bodily Pain scale and does not change any statistical results (including *p*‐values), because all analyses were performed using appropriately scored SF‐36 Bodily Pain values. We have revised the relevant statements in the Methods, Results, Discussion, and Supporting Information to ensure that the interpretation of SF‐36 Bodily Pain scores is accurate and consistent throughout.

## Methods

### Psychoneurological (PN) Symptoms

Bodily pain was measured using the SF‐36 [2]. For this subscale, higher scores indicate less bodily pain (i.e., better health status), whereas lower scores indicate more bodily pain.

In contrast, higher scores on the respective questionnaires indicate more pronounced symptomatology for anxiety, depression, fatigue, neuropathic pain, and sleep disturbance. The directions and ranges of scores are available in Table S3.

## Results

### Symptom Phenotypes of Each Cluster

Bodily pain was greatest (lowest SF‐36 Bodily Pain scores) in Cluster 1 and least (highest scores) in Cluster 5 (Kruskal–Wallis test, *p* < 0.0001) (Figure 5B).

## Discussion

Cluster 5 had graduate educational degrees, the highest physical function, dispositional optimism, overall social support, and the mildest symptomatology. On the contrary, participants in Cluster 1 were mostly unemployed or homemakers, mostly earning < $15 K, with the lowest educational attainment and physical function, but the greatest bodily pain, fatigue, and neuropathic pain.

## Supplementary Materials

**Table S3**. Symptom phenotypes in each cluster group.

**Table:** 

		Direction higher is	Range of scores	Central tendency (*N* = 414)	Cluster					*p*‐value	Test
					1 (*n* = 59)	2 (*n* = 59)	3 (*n* = 102)	4 (*n* = 92)	5 (n = 102)		
PROMIS	Anxiety [square root]	Worse	8–40 [2.8–6.3]	4.3 (0.8)	4.9 (0.6)	5.2 (0.5)	4.4 (0.5)	3.6 (0.5)	3.9 (0.7)	<0.001	a
	Depression	Worse	8–40	15 (11.8)	22 (9)	28 (6.5)	16.5 (7)	11 (6)	10 (4)	<0.001	k
	Fatigue	Worse	8–40	24.2 (8.5)	33.6 (5.7)	30.0 (6.3)	26.5 (5.8)	21.3 (6.7)	15.7 (5.0)	<0.001	a
	Neuropathic Pain	Worse	5–25	8 (7)	19 (6)	9 (3.5)	7 (5)	9 (7)	5 (2)	<0.001	k
	Sleep Disturbance	Worse	8–40	23.9 (8.2)	30.1 (7.4)	30.0 (6.2)	27.3 (6.4)	18.0 (5.6)	18.7 (6.1)	<0.001	a
SF‐36	Bodily Pain	Better	0–100	57.5 (45)	32.5 (11.2)	45 (35)	67.5 (32.5)	45 (25)	90 (12.5)	<0.001	k

a, analysis of variance, the values are shown as mean (standard deviation); k, Kruskal‐Wallis test, the values are shown as median (interquartile range); PROMIS, Patient‐Reported Outcomes Measurement Information System; SF‐36, 36‐Item Short‐Form Health Survey.

We apologize for this error.
