# Supplementary material for: Characterization of cancer survivors clustered by subjective and objective cognitive function scores
Source: Cancer Med. 2024 Jun 21;13(12):e7255. doi: 10.1002/cam4.7255 (PMC11192644; doi:10.1002/cam4.7255)
Supplement: Supplementary file 1 — Data S1: [file CAM4-13-e7255-s002.docx]

**SUPPLEMENTARY MATERIALS**

**Methods**

Cognitive Function

**Subjective Cognitive Function Assessment.** Perceived cognitive impairment was assessed using the Patient-Reported Outcomes Measurement Information System (PROMIS) version 1.0 short-form subscales of Cognitive Abilities and Cognitive Concerns, each containing eight items. The Cognitive Abilities items target positive self-assessments of cognitive functioning such as “My memory has been as good as usual” and “I have been able to concentrate.” Higher scores indicate higher cognitive ability. The Cognitive Concerns items are worded negatively and express concerns in the same areas. Two examples are “My thinking has been slow” and “I have had trouble shifting back and forth between different activities that require thinking.” Items on both subscales use a 5-point rating from “not at all” to “very much.” Higher scores indicate higher cognitive concerns. Items are summed to create a total score for each subscale. These relatively short scales were derived from an intensive item analysis using Item Response Theory and qualitative analysis to produce short but reliable measures. The directions and ranges of scores are available in Supplementary Table S1.

**Objective Cognitive Function Assessment.** A neuropsychological assessment was conducted online (remotely) using CANTAB, Cambridge Cognition®, a reliable and valid program used in several clinical populations, including cancer survivors, to assess cognitive performance. The specific CANTAB, Cambridge Cognition® cognitive domains selected for testing in the parent study were common areas of concern for cancer survivors, including (1) visuospatial working memory capacity measured by the CANTAB - spatial span (SSPFSL); (2) visual episodic memory and new learning measured by the CANTAB paired associates learning (PALTE); (3) working memory and executive function measured by the CANTAB - spatial working memory (SWMBE468); and (4) sustained attention measured by the CANTAB - rapid visual information processing (RVPA and RVPPFA). The directions and ranges of scores are available in Supplementary Table S1.

Physical Function, Affects, Personality, and Social Support

Physical function was assessed using the 10-item Physical Function subscale of the PROMIS short form, with responses rated on a 5-point Likert scale from 0 to 4. Higher scores indicated worse physical function.

Positive and negative affects were evaluated using the Positive and Negative Affect Schedule (PANAS), which consists of ten pleasant and unpleasant affective states. Participants rated the extent to which they experienced each mood during the past week on a 5-point scale. Total scores for the positive and negative affect subscales ranged from 0 to 40.

Dispositional optimism was assessed using the 10-item Life Orientation Test-Revised. Responses were rated on a 5-point Likert scale ranging from strongly disagree to strongly agree, with higher scores indicating greater optimism.

Perceived social support was evaluated using the Medical Outcomes Study–Social Support Survey (MOS-SSS). Participants rated the availability of someone to listen, confide in, share worries with, and understand their problems. Scores for MOS-SSS social support ranged from 0 to 100, with higher scores indicating greater perceived emotional support.

The directions and ranges of scores are available in Supplementary Table S2.

Psychoneurological Symptoms

Anxiety, depression, fatigue, neuropathic pain, and sleep disturbance were assessed using PROMIS short forms. The PROMIS measures are standardized and widely validated for the assessment of symptoms across clinical populations. The scales have a 5-point Likert scale from 0 to 4, with higher scores indicating worse symptomology (greater depression, anxiety, fatigue, and sleep disturbance). Bodily pain was measured by the 36-Item Short-Form Health Survey (SF-36) bodily pain subscale, consisting of two-items. The SF-36 has been established as a comprehensive measure of general health that has shown reliability and validity in various populations, including cancer patients.

The directions and ranges of scores are available in Supplementary Table S3.
