# Supplementary material for: Characterization of cancer survivors clustered by subjective and objective cognitive function scores
Source: Cancer Med. 2024 Jun 21;13(12):e7255. doi: 10.1002/cam4.7255 (PMC11192644; doi:10.1002/cam4.7255)
Supplement: Supplementary file 2 — Data S2: [file CAM4-13-e7255-s001.docx]

**SUPPLEMENTARY MATERIALS**

**Results**

**Figure S1.** Social support sub-scales

The Kruskal-Wallis test followed by pairwise Wilcoxon’s rank sum tests was applied.

| **Table S1.** Subjective and objective cognitive function scores in each cluster group | | | | | | | | | | | |
| --- | --- | --- | --- | --- | --- | --- | --- | --- | --- | --- | --- |
|  | | Direction Higher is: | Range of Scores | Central Tendency  (N = 414) | Cluster | | | | | *p*-value | Test |
|  |  |  |  |  | 1  (n = 59) | 2  (n = 59) | 3  (n = 102) | 4  (n = 92) | 5  (n = 102) |  |  |
| PROMIS | Cognitive Abilities | Better | 8–40 | 20.9 (6.8) | 15.3 (4.9) | 16.2 (4.8) | 20.4 (5.6) | 24.3 (6.4) | 24.3 (6.3) | < .001 | a |
| PROMIS | Cognitive Concerns | Worse | 8–40 | 27 (11) | 34 (10.5) | 32 (10) | 26 (8) | 24 (11) | 23 (9) | < .001 | k |
| CANTAB | SSPFSL | Better | 2–9 | 6 (2) | 6 (1) | 6 (1.5) | 6 (2) | 6 (2) | 6 (1) | .25 | k |
|  | PALTE [square root] | Worse | 0–68 [0–8.25] | 2.9 (1.3) | 3.2 (1.2) | 2.9 (1.3) | 2.8 (1.2) | 2.9 (1.3) | 2.8 (1.3) | .38 | a |
|  | SWMBE468 | Worse | 0–153 | 11.5 (18) | 15 (13) | 14 (18) | 8 (17) | 14 (15.2) | 6.5 (15.8) | .02 | k |
|  | RVPA | Better | 0–1 | 0.9 (0.06) | 0.90(0.05) | 0.9 (0.07) | 0.9 (0.06) | 0.9 (0.06) | 0.9 (0.06) | .05 | k |
|  | RVPPFA [⨉ 1000] | Worse | 0–1 [0–1000] | 4.0 (6.2) | 4.1 (8.3) | 4 (4.8) | 3.9 (6.5) | 4 (5.8) | 3.9 (4.8) | .43 | k |
| a, analysis of variance, the values are shown as mean (standard deviation); k, Kruskal-Wallis test, the values are shown as median (interquartile range); PROMIS, Patient-Reported Outcomes Measurement Information System; SSPFSL, Visuospatial working memory capacity using the CANTAB-spatial span; PALTE, Visual episodic memory and new learning using the CANTAB paired associates learning; SWMBE468, Working memory and executive function via CANTAB-spatial working memory; RVPA and RVPPFA, Sustained attention via CANTAB-rapid visual information processing | | | | | | | | | | | |

| **Table S2.** Physical function, affects, personality, and social support in each cluster group | | | | | | | | | | | |
| --- | --- | --- | --- | --- | --- | --- | --- | --- | --- | --- | --- |
|  | | Direction Higher is: | Range of Scores | Central Tendency  (N = 414) | Cluster | | | | | *p*-value | Test |
|  |  |  |  |  | 1  (n = 59) | 2  (n = 59) | 3  (n = 102) | 4  (n = 92) | 5  (n = 102) |  |  |
| Physical Functioning-10 | Physical Function | Better | 0–100 | 80 (35) | 45 (42.5) | 70 (35) | 80 (30) | 75 (26.2) | 95 (15) | < .001 | k |
| Positive and Negative Affect Schedule | Positive | Better | 10–50 | 26.4 (6.7) | 22.3 (5.7) | 20.6 (5.2) | 25.6 (5.6) | 28.9 (6.2) | 30.6 (5.5) | < .001 | a |
|  | Negative | Worse | 10–50 | 21.6 (7.3) | 26.6 (7.6) | 29.8 (6.5) | 22.5 (5.4) | 16.7 (4.4) | 17.3 (4.4) | < .001 | a |
| Life Orientation Test - Revised | | Better | 0–24 | 14.8 (4.8) | 11.9 (5.0) | 10.4 (5.3) | 14.9 (4.0) | 16.8 (3.4) | 17.0 (3.3) | < .001 | a |
| MOS-SSS | Emotional/informational support | Better | 8–40 | 26.7 (8.2) | 23 (14.5) | 21 (13) | 27.5 (13.8) | 29 (12.2) | 31.5 (11) | < .001 | k |
|  | Tangible support | Better | 4–20 | 16 (8) | 15 (8) | 16 (9) | 16 (6.8) | 16 (6.3) | 19 (7) | < .001 | k |
|  | Affectionate support | Better | 3–15 | 14 (6) | 12 (7) | 11 (8.5) | 13 (4) | 14 (3) | 15 (4) | < .001 | k |
|  | Positive social interaction | Better | 3–15 | 12 (6) | 11 (6) | 9 (6) | 11 (4) | 12 (5.3) | 13.5 (3) | < .001 | k |
|  | Overall social support | Better | 5–95 | 72 (28) | 62 (23.5) | 58 (33) | 71 (27) | 75 (21.5) | 81.5 (25) | < .001 | k |
| a, analysis of variance, the values are shown as mean (standard deviation); k, Kruskal-Wallis test, the values are shown as median (interquartile range); PROMIS, Patient-Reported Outcomes Measurement Information System; PNAS, Positive and Negative Affect Schedule; MOS-SSS, Medical Outcomes Study–Social Support Survey | | | | | | | | | | | |

| **Table S3.** Symptom phenotypes in each cluster group | | | | | | | | | | | |
| --- | --- | --- | --- | --- | --- | --- | --- | --- | --- | --- | --- |
|  | | Direction Higher is: | Range of Scores | Central Tendency  (N = 414) | Cluster | | | | | *p*-value | Test |
|  |  |  |  |  | 1  (n = 59) | 2  (n = 59) | 3  (n = 102) | 4  (n = 92) | 5  (n = 102) |  |  |
| PROMIS | Anxiety [square root] | Worse | 8–40 [2.8–6.3] | 4.3 (0.8) | 4.9 (0.6) | 5.2 (0.5) | 4.4 (0.5) | 3.6 (0.5) | 3.9 (0.7) | < .001 | a |
|  | Depression | Worse | 8–40 | 15 (11.8) | 22 (9) | 28 (6.5) | 16.5 (7) | 11 (6) | 10 (4) | < .001 | k |
|  | Fatigue | Worse | 8–40 | 24.2 (8.5) | 33.6 (5.7) | 30.0 (6.3) | 26.5 (5.8) | 21.3 (6.7) | 15.7 (5.0) | < .001 | a |
|  | Neuropathic Pain | Worse | 5–25 | 8 (7) | 19 (6) | 9 (3.5) | 7 (5) | 9 (7) | 5 (2) | < .001 | k |
|  | Sleep Disturbance | Worse | 8–40 | 23.9 (8.2) | 30.1 (7.4) | 30.0 (6.2) | 27.3 (6.4) | 18.0 (5.6) | 18.7 (6.1) | < .001 | a |
| SF-36 | Bodily Pain | Worse | 0–100 | 57.5 (45) | 32.5 (11.2) | 45 (35) | 67.5 (32.5) | 45 (25) | 90 (12.5) | < .001 | k |
| a, analysis of variance, the values are shown as mean (standard deviation); k, Kruskal-Wallis test, the values are shown as median (interquartile range); PROMIS, Patient-Reported Outcomes Measurement Information System; SF-36, 36-Item Short-Form Health Survey | | | | | | | | | | | |
